# Preparation of aluminium-hydroxide-modified diatomite and its fluoride adsorption mechanism

**DOI:** 10.1038/s41598-023-30901-8

**Published:** 2023-03-08

**Authors:** Biao Xiang, Jiaxi Tang, Xiaojie Feng, Yongle Zhu, Yu Li, Ting Tan

**Affiliations:** College of Environmental Science and Engineering, Liaoning Technical University, Fuxin, 123000 China

**Keywords:** Ecology, Environmental sciences

## Abstract

As the current excessive accumulation of fluoride (F^−^) in the environment can be hazardous to human health, it is essential to remove fluoride from wastewater. In this study, diatomite (DA) was used as a raw material and modified using aluminum hydroxide (Al-DA) for use in the adsorption of F^−^ from water bodies. SEM, EDS, XRD, FTIR, and Zeta potential characterization analyses were carried out; adsorption tests and kinetic fitting were performed, and the effects of pH, dosing quantity, and presence of interfering ions on the adsorption of F^−^ by the materials were investigated. The results show that the Freundlich model effectively describes the adsorption process of F^−^ on DA, which therefore involves adsorption-complexation interactions; however, the Langmuir model effectively describes the adsorption process of F^−^ on Al-DA, corresponding to unimolecular layer adsorption mainly via ion-exchange interactions, that is, adsorption is dominated by chemisorption. Aluminum hydroxide was shown to be the main species involved in F^−^ adsorption. The efficiency of F^−^ removal by DA and Al-DA was over 91% and 97% for 2 h, and the adsorption kinetics were effectively fit by the quasi-secondary model, suggesting that chemical interactions between the absorbents and F^−^ control the adsorption process. The adsorption of F^−^ was highly dependent on the pH of the system, and the maximum adsorption performance was obtained at pH 6 and 4. The optimal dosage of DA and Al-DA was 4 g/L. Even in the presence of interfering ions, the removal of F^−^ on Al-DA reached 89%, showing good selectivity. XRD and FTIR studies showed that the mechanism of F^−^ adsorption on Al-DA involved ion exchange and the formation of F–Al bonds.

## Introduction

Fluoride is a form of monoatomic F that is accepted as a micronutrient because of its ability to support dental development^1^. Therefore, F^−^ intake within permissible limits is beneficial for healthy production and maintenance of healthy teeth and bones in humans^2^. Conversely, chronic intake of high levels of fluoride can lead to many health problems, such as dental and bone fluorosis^3^. Fluoride concentrations above 10.0 mg/L can lead to serious diseases, such as hypertension, neurological disorders, and even cancer^4^. Therefore, the World Health Organization recommends a fluoride concentration of 1.5 mg/L in drinking water as a safety guideline^5^. As fluoride is currently widely used in various industries, fluorinated wastewater is increasingly being discharged from many engineering processes, such as metalworking, glass, enamel, brick, semiconductor manufacturing, coal-fired power plants, electroplating, rubber, and fertilizer industries^6–9^. Globally, more than 200 million people are reported to drink water with fluoride concentrations above 1.5 mg/L and suffer from various forms of fluorosis^10^. These problems are particularly severe in developing countries, such as Kenya, India, Iran, and China^11–15^. Therefore, the removal of fluoride from water bodies has become a problem that needs to be solved urgently.

Several techniques for the removal of fluoride ions from water have been reported to date, including precipitation, electrosorption, membrane separation, the use of ion exchange resins, coagulation, and adsorption^16–21^. Among these methods, adsorption is considered to be a promising technique due to its ease of operation and economy; thus, it is important to identify effective adsorbents for fluoride removal^22^. Current adsorbents used for fluoride removal include metal–organic frameworks (MOFs), hydroxyapatite microspheres, zinc-magnesium–aluminum ternary oxide microspheres, engineered biochar, diatomite^23–27^, Among these adsorbents, diatomite (DA) is an amorphous naturally occurring material with unique physical and chemical properties, such as high permeability and porosity, a small particle size, high adsorption capacity, low thermal conductivity and density, and a high surface area, and is widely used for adsorption of pollutants from water^28^. Xu^29^ investigated the use of natural diatomite for fluoride removal and found that using 40–60 g/L of diatomaceous earth adsorbed 82% of fluoride at an initial concentration of 5 mg/L at pH 5. In recent years, scholars have been working on modifying adsorbents to improve performance. Liu^30^ prepared diatomite-based adsorbents for mercury removal using aqueous impregnations of diatomite and different active substances (CuCl_2_, CuBr_2_, NaI, NaBr, NaCl, KI, KBr, and KCl) and achieved 91% adsorption. Therefore, we have continued to search for an effective modifier for these adsorbents. According to Pearson's hard-soft acid–base (HSAB) theory, the high electronegativity and small size of fluoride ions (hard Lewis bases) should result in a strong affinity for Al(III) (hard Lewis acids)^6^. It is speculated that the affinity of aluminium hydroxide for fluoride should make material such as diatomite modified with aluminum hydroxide a good adsorbent for fluoride removal.

Therefore, this study aimed to modify DA with aluminium hydroxide (Al-DA) and compare the adsorption potential with that of DA for F^−^ removal from water. Scanning electron microscopy (SEM), energy spectroscopy (EDS), X-ray diffraction spectroscopy (XRD), Fourier transform infrared spectroscopy (FTIR), and Zeta potential were used to study the properties of the prepared materials. The effects of various parameters (solution pH, adsorbent dose, and presence of interfering ions) on the adsorption process were evaluated, and the adsorption mechanism was elucidated to provide a theoretical basis for the treatment of fluoridated waters.

## Materials and methods

### Chemical reagents and materials

The diatomite (DA) used in the experiments was purchased from Jiangsu Chengbo Environmental Technology Co., Ltd. in China; aqueous fluoride (F^−^) was simulated using high-purity sodium fluoride purchased from Tianjin Windship Chemical Reagent Technology Co. Deionized water was used in the experiments; sodium citrate dihydrate and sodium chloride were purchased from Tianjin Guangfu Science and Technology Development Co. Sodium hydroxide was purchased from Tianjin New Technology Industrial Park, Tianjin, China, and aluminium chloride was purchased from Shanghai Zhangyun Chemical Co. Sodium bicarbonate and anhydrous sodium carbonate were purchased from Liaoning Quanrui Reagent Co., Ltd. in China; sodium nitrate and anhydrous sodium sulfate were purchased from Huadong Reagent Factory in Shenyang, China.

### Preparation and characterization of Al-DA

Thirty grams of DA were packed into a 1 L plastic bottle, to which 100 mL of 1 mol/L AlCl_3_-6H_2_O and 3 mol/L NaOH were added. The bottle was placed in a shaker at 200 rpm for 2 h at room temperature. After equilibration, the mixture was centrifuged, and the recovered solid was ladled into a 1 L bottle containing distilled water. The solid was washed with distilled water and centrifuged repeatedly until the pH of the supernatant was 6. The solid residue was dried in an oven at 105 °C for 12 h, cooled in a desiccator, passed through an 80-mesh sieve, and stored until use.

The microscopic morphology and surface characteristics of the materials before and after adsorption were observed by scanning electron microscopy (SEM, JSM-7610FPlus, Japan); the surface chemical composition of the materials before and after adsorption was determined by energy spectrometry (EDS, ULTIM MAX 40, Oxford, UK); the composition and crystal structure of the materials before and after adsorption were characterized by X-ray diffraction (XRD, Bruker D8 Advance); and a Fourier transform infrared spectrometer (FT-IR, Shimadzu-IRTracer-100, Japan) was used to identify changes in the surface structural groups resulting from adsorption. Liquid Zeta particle size/potential analysis (UK -Malvern-Zetasizer Nano S90) was used to analyze the zeta potential of materials at different pH values.

### Effect of pH on the adsorption effect

Take 25 mL of F^−^ solution with a concentration of 100 mg/L and add it to a 50 mL centrifuge tube, and the pH was adjusted to 3.0, 4.0, 5.0, 6.0, 7.0, and 8.0, respectively. 0.10 g of two adsorbent materials were added, and the adsorption experiments were carried out in a constant temperature shaker at 25 °C. Take it out after 120 min of adsorption. After centrifugation at 4000 r/min for 10 min, 10 mL of supernatant was centrifuged through a 0.45 μm filter membrane, and the remaining F^−^ mass concentration in the solution was determined by a PXS-270 fluoride ion selective electrode. Each experimental treatment was repeated three times.

### Effect of dosage on the adsorption effect

The two adsorbent materials were weighed at 0.04, 0.08, 0.10, 0.15, 0.20, and 0.30 g in 50 mL centrifuge tubes, and 25 mL of a mass concentration of 100 mg/L and a pH of 6 F^−^ a solution was added to each tube for adsorption experiments at 25 °C in a constant temperature shaker. Take it out after 120 min of adsorption. After centrifugation at 4000 r/min for 10 min, 10 mL of the supernatant was centrifuged through a 0.45 μm filter membrane, and then the remaining F^−^ mass concentration in the solution was measured. Each experimental treatment was repeated three times.

### Effect of coexisting anions on the adsorption of F^−^

To investigate the effect of coexisting anions on adsorption, competing ions (Cl^−^, NO_3_^−^, CO_3_^2−^, SO_4_^2−^, HCO_3_^−^, and CO_3_^2−^) were used in experiments at pH 4, 5, 6, and 7 while maintaining an ion concentration of 100 mg/L, a temperature of 25 °C, a contact time of 2 h, an adsorbent dose of 4.0 g/L.

The removal efficiency (%) and adsorption capacity (Q_e_) were measured using the following equations:

1 $${\text{Removal~}}\;{\text{efficiency}}\;{\text{ = }}\;\frac{{{\text{C}}_{{\text{0}}} - {\text{C}}_{{\text{e}}} }}{{{\text{C}}_{{\text{0}}} }}\; \times \;100\%$$

2 $$q_{{\text{e}}} \;{\text{ = }}\;\frac{{\left( {{\text{C}}_{{\text{0}}} - {\text{C}}_{{\text{e}}} } \right){\text{V}}}}{{\text{m}}}$$

where ${\text{C}}_{0}$ is the initial mass concentration of F^−^ in the solution (mg/L), ${\text{C}}_{\text{e}}$ is the mass concentration of F^−^ of the solution at adsorption equilibrium (mg/L), respectively; V is the volume of the solution (L); m is the material mass (g); and q_e_ is the adsorption capacity of F^−^ at the adsorption equilibrium (mg/g).

## Results and discussion

### Scanning electron microscopy and energy spectrum analysis

**Figure 1:**
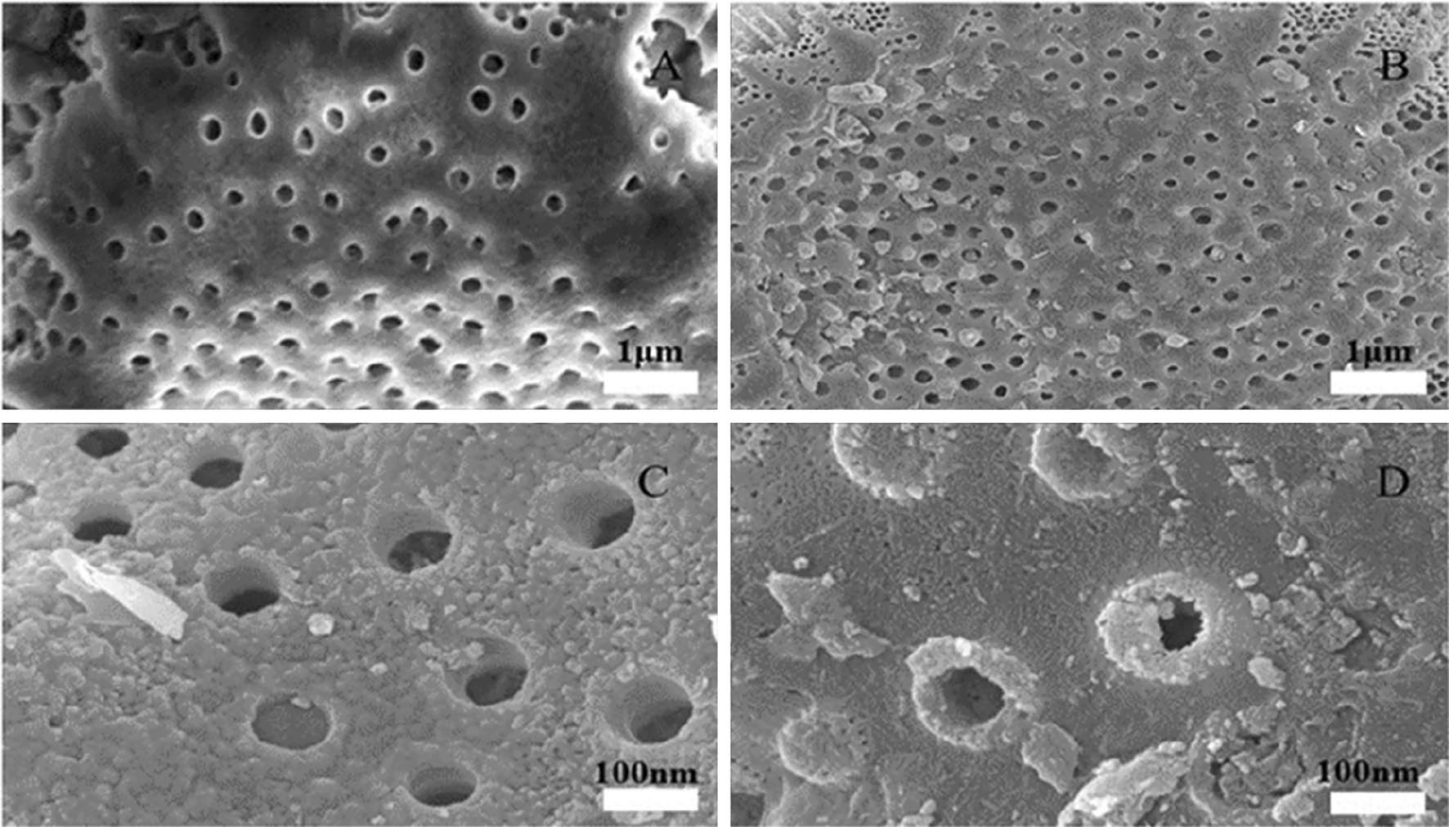
SEM images of DA and Al-DA before and after adsorption. (**A**) Before DA adsorption. (**B**) After DA adsorption. (**C**) Before Al-DA adsorption. (**D**) After Al-DA adsorption.

**Figure 2:**
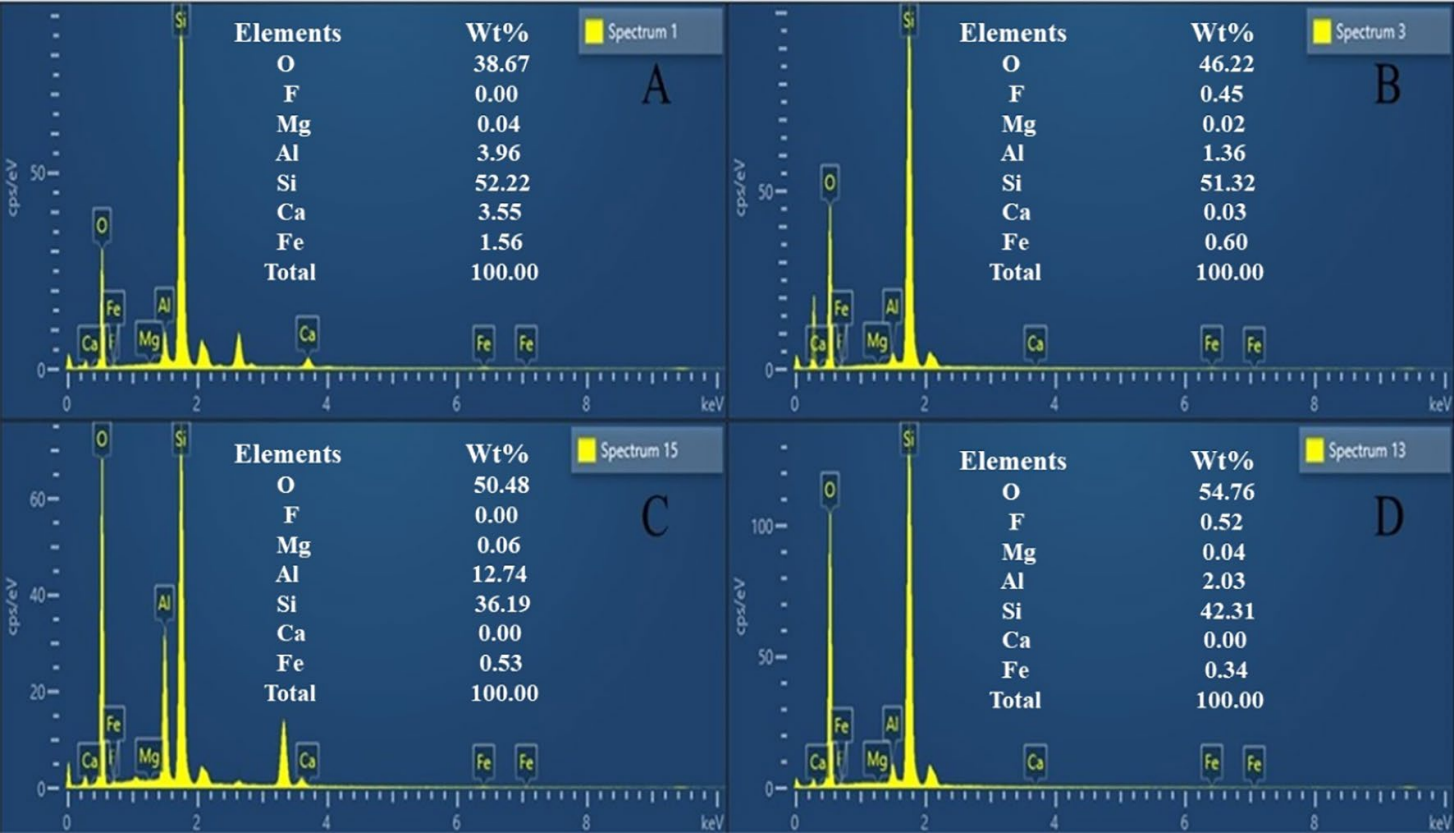
EDS graphs of DA and Al-DA before and after adsorption. (**A**) Before DA adsorption. (**B**) After DA adsorption. (**C**) Before Al-DA adsorption. (**D**) After Al-DA adsorption.

The SEM images show the morphological structures of DA and Al-DA before and after adsorption (Fig. 1). DA and Al-DA have disk-like microstructures^29^ with sur-faces containing both large and small pores, that is, DA and Al-DA have unique multi-level pore structures. The main component of DA and Al-DA is silica, which has a large specific surface area, good thermal stability, and is a natural green material for use as a water treatment agent with a porous structure^31^. The micrographs show that before adsorption, the DA surface is smooth with a distinct pore structure, whereas modification with aluminium hydroxide makes DA coarse and loose because of the formation of amorphous aluminium hydroxide colloids^32^. After adsorption, the surface pore structure is covered over for DA and completely covered over for Al-DA, which indicates that F^−^ reacts with Al^3+^ to form nanoscale precipitates^22^. The results of the EDS analysis (Fig. 2) show that the content of elemental Al increased from 3.96 to 12.74% after DA was modified with aluminium hydroxide, indicating that Al adhered effectively to the modified DA surface. After adsorption, the content of elemental Al decreased from 3.96 to 1.36% for DA and from 12.74 to 2.03% for Al-DA, which fully confirmed that fluorine preferentially combined with Al to form aluminium precipitates during adsorption, thereby decreasing the Al content.

### XRD analysis

**Figure 3:**
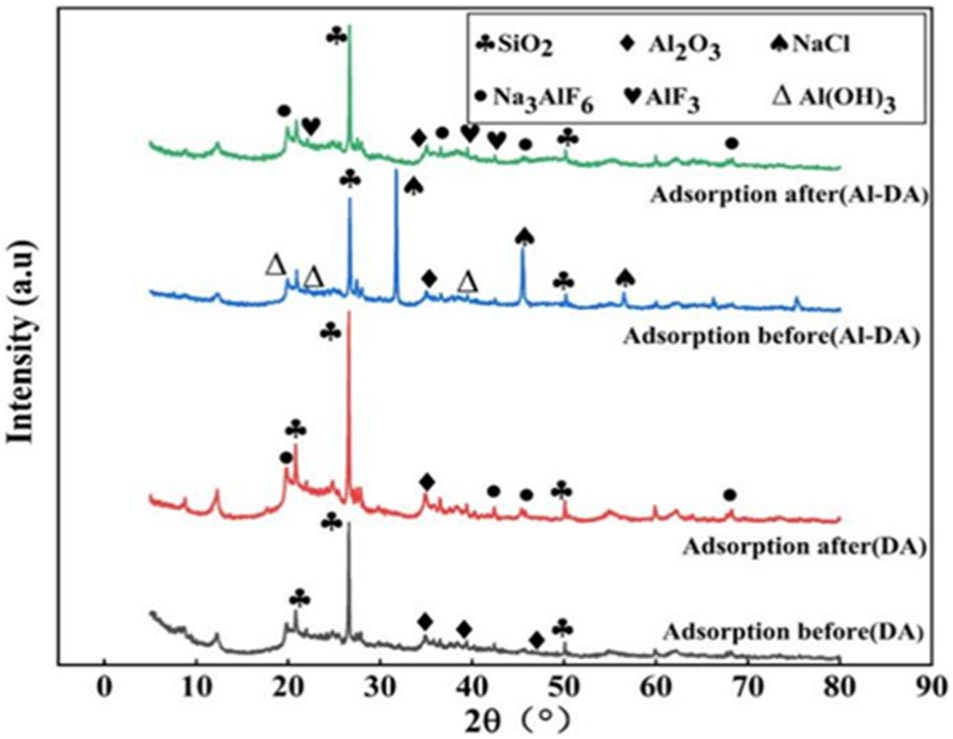
XRD patterns of DA and Al-DA before and after adsorption.

The surface mineral composition and crystallinity of the materials before and after adsorption were analyzed by XRD (Fig. 3). In the DA and Al-DA patterns, the wide diffraction peaks at approximately 22.0°, 26.0°, and 50.0° mainly correspond to amorphous SiO_2_, and the diffraction peak at approximately 35° mainly corresponds to amorphous Al_2_O_3_, indicating that the material is polycrystalline^29^. It has been re-ported that amorphous materials may be good adsorbents because of a large specific surface area and numerous active sites^33^. Many Al(OH)_3_ peaks and NaCl peaks appear in the XRD pattern of Al-DA, indicating the successful modification of DA by aluminium hydroxide. After adsorption, Na_3_AlF_6_ peaks appear in the DA pattern, and Na_3_AlF_6_ and AlF_3_ peaks appear in the Al-DA pattern, whereas the characteristic peaks of NaCl are absent in the Al-DA pattern, which indicates the participation of NaCl in the adsorption process. It has been demonstrated that in the presence of excess sodium fluoride in the reaction solution, the generated aluminium fluoride combines with sodium fluoride to form a NaAlF_4_ intermediate, which is subsequently converted to cryolite complexes by further adsorption of sodium fluoride^34^. This result confirms the XRD mapping results.

### Infrared analysis

**Figure 4:**
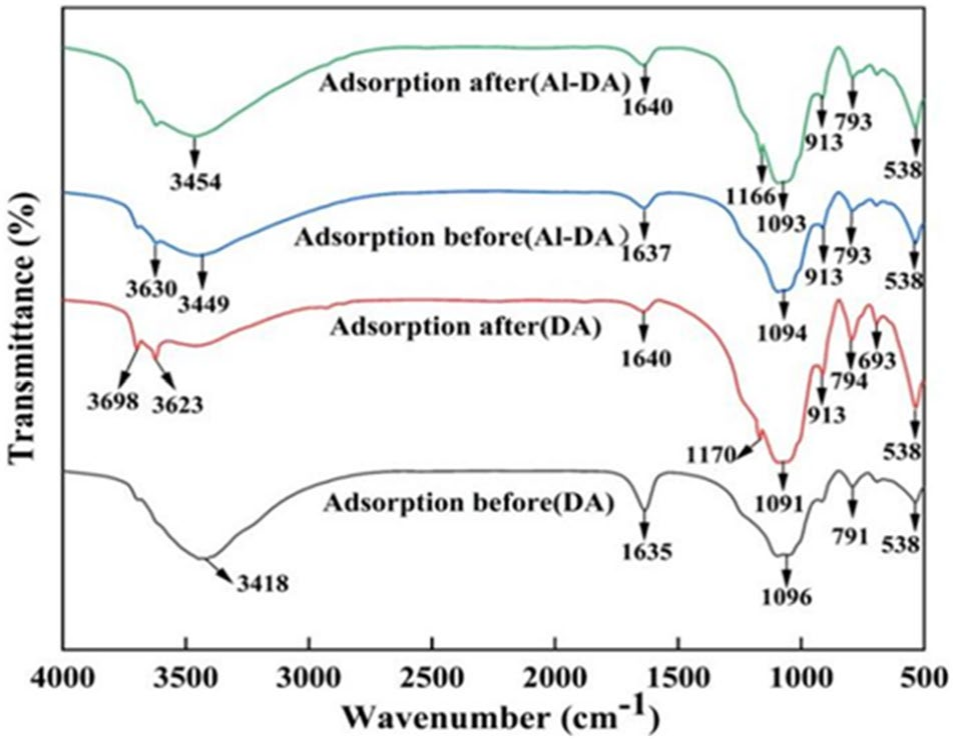
FTIR spectra of DA and Al-DA before and after adsorption.

Figure 4 shows the FTIR spectra of DA and Al-DA before and after adsorption: peaks at 3418, 1635, 1096, 791, and 538 cm^−1^ appear in the spectrum of DA spectrum before adsorption, and peaks at 3630, 3449, 1637, 1094, 913, 793, and 538 cm^−1^, appear in the Al-DA spectrum before adsorption. The strong and broad band centered at 3418 cm^−1^ is due to the stretching vibration of the adsorbed water hydroxyl group (O–H) and the surface hydroxyl group, the vibrational peak at approximately 1635 cm^−1^ is probably from bound water or the surface hydroxyl group; the peaks at 1096 cm^−1^ and 538 cm^−1^ correspond to siloxane groups (Si–O–Si–) and an Al–O absorption band, respectively; and the strong oscillations at 791 cm^−1^ may be attributed to inorganic Al salts^35–37^. The original absorption peak in the DA spectrum is shifted in the spectrum of DA modified with aluminium hydroxide, confirming the successful modification of DA. The shift of the band at 3418 cm^−1^ in the DA spectrum to a higher frequency at 3623 cm^−1^ in the DA spectrum after fluoride absorption is caused by fluoride bonding and has been previously reported^38^. Another noticeable change in the spectra of DA and Al-DA before and after adsorption is the increase or decrease in the intensity of bending vibrations of specific peaks because the highly electronegative fluoride may have an inductive effect on the respective groups that leads to a blueshift, and the formation of hydrogen bonds leads to a redshift and broadening of the spectral band. The shifts and changes of these peaks indicate the interaction of fluoride with the respective groups^29^. The new peak at approximately 1170 cm^−1^ in the spectra of DA and Al-DA with adsorbed fluoride may be due to the formation of Al-F bonds^6^. The IR spectra show that the formation of a new bonding electronic structure by surface complexation with F^−^ is one of the main mechanisms for the adsorption of F^−^.

### Zeta potential analysis

**Figure 5:**
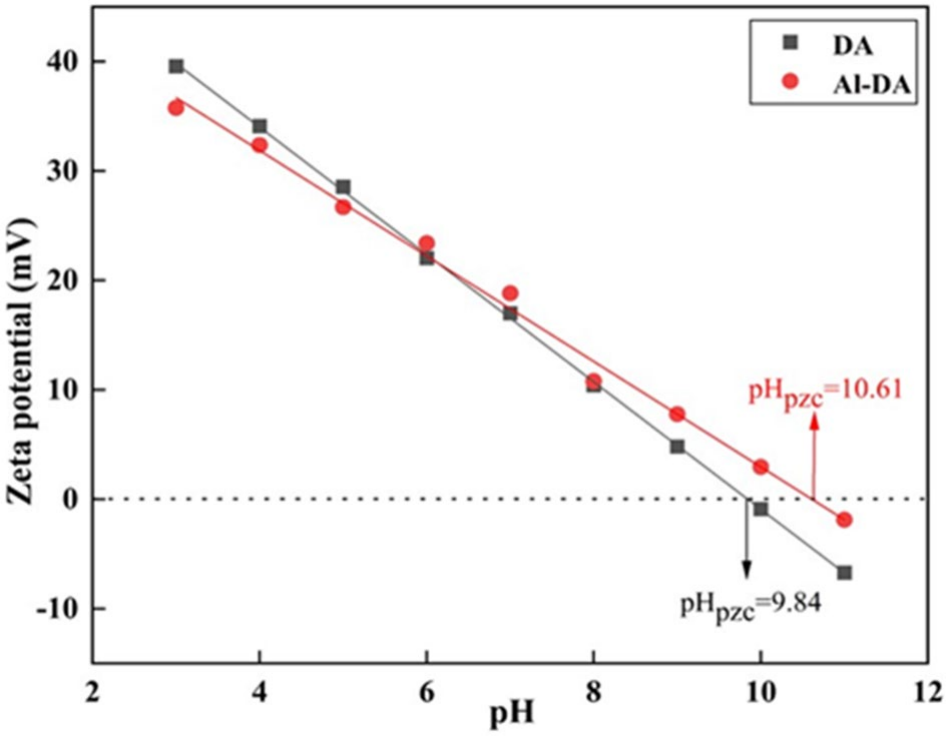
Zeta potential of DA and Al-DA at different pH values.

The zeta potential of the material surface plays a very key role in the adsorption process, which reflects the surface charge properties of the material under different pH conditions, and also reflects the surface properties of the material. To obtain the zero charge point of the material, we studied the potential change of the material under different pH values. The results are shown in Fig. 5. In the range of pH 3–11, the zeta potential of the two adsorbents decreased linearly with the increase in pH, and the pH_zpc_ of DA and Al-DA were 9.84 and 10.61, respectively. When pH < pH_zpc_, the material surface is positively charged, and high electronegativity fluorine ions should be adsorbed to the material's positively charged surface. On the other hand, when pH value > pH_zpc_, the ad-sorbent surface is electronegative, resulting in electrostatic repulsion between the ad-sorbent surface and electronegative fluoride ions. Therefore, at a higher pH value, the adsorption of fluoride ions is reduced^39^. Therefore, for the negatively charged fluoride ion, the surface of the two adsorbents is positively charged in a wide pH range, which can show a good adsorption effect, and Al-DA is better. Owing to the modification-induced formation of new aluminosilicate compounds on the diatomite surface, the pH_PZC_ of Al-DA is shifted to higher values. The partial substitution of Al atoms for Si atoms in silicates, owing to their different valences, generates an excess negative charge, which is compensated by the introduction of Na^+^, K^+^, Mg^2+^, or Ca^2+^ cations into the aluminosilicate crystal lattice. An increase in the Al/Si ratio leads to a decrease in the aluminosilicate ion sizes and an increase in the total negative charge per aluminum atom; as a result, the sorbent surface in solution adsorbs a larger number of protons and the pH_PZC_ is shifted to higher values^34^.

### Adsorption isotherms

To better understand the behavior and capacity of the adsorbents for fluoride, the Langmuir and Freundlich isotherm models were used to fit the experimental data. The Langmuir model is based on the assumption of uniform monolayer adsorption with no interactions between adsorbed species. By contrast, the Freundlich model is an empirical equation that describes a heterogeneous system with multilayer adsorption^38^ and the formula is as follows:

3 $$\begin{array}{c}{\text{q}}_{\text{e}} = \frac{{\text{K}}_{\text{L}}{{\text{Q}}}_{\text{m}}{{\text{C}}}_{\text{e}}}{\text{1} + {\text{K}}_{\text{L}}{{\text{C}}}_{\text{e}}}\end{array}$$

4 $$\begin{array}{c}{\text{q}}_{\text{e}} = {\text{K}}_{\text{F}}{{\text{C}}}_{\text{e}}^{\frac{1}{{\text{n}}}}\left(4\right)\end{array}$$

where C_e_ is the mass concentration of F^−^ in the solution at adsorption equilibrium (mg/L); q_e_ is the quantity of F^−^ adsorbed at equilibrium (mg/g); Q_m_ is the maximum saturated adsorbed quantity (mg/g); K_L_ is a constant in Langmuir’s equation related to the heat of adsorption; K_F_ is a constant in the Freundlich equation related to the ad-sorption strength; and n > 1 for preferential adsorption, n = 1 for linear adsorption for, and n < 1 for nonpreferential adsorption.

**Figure 6:**
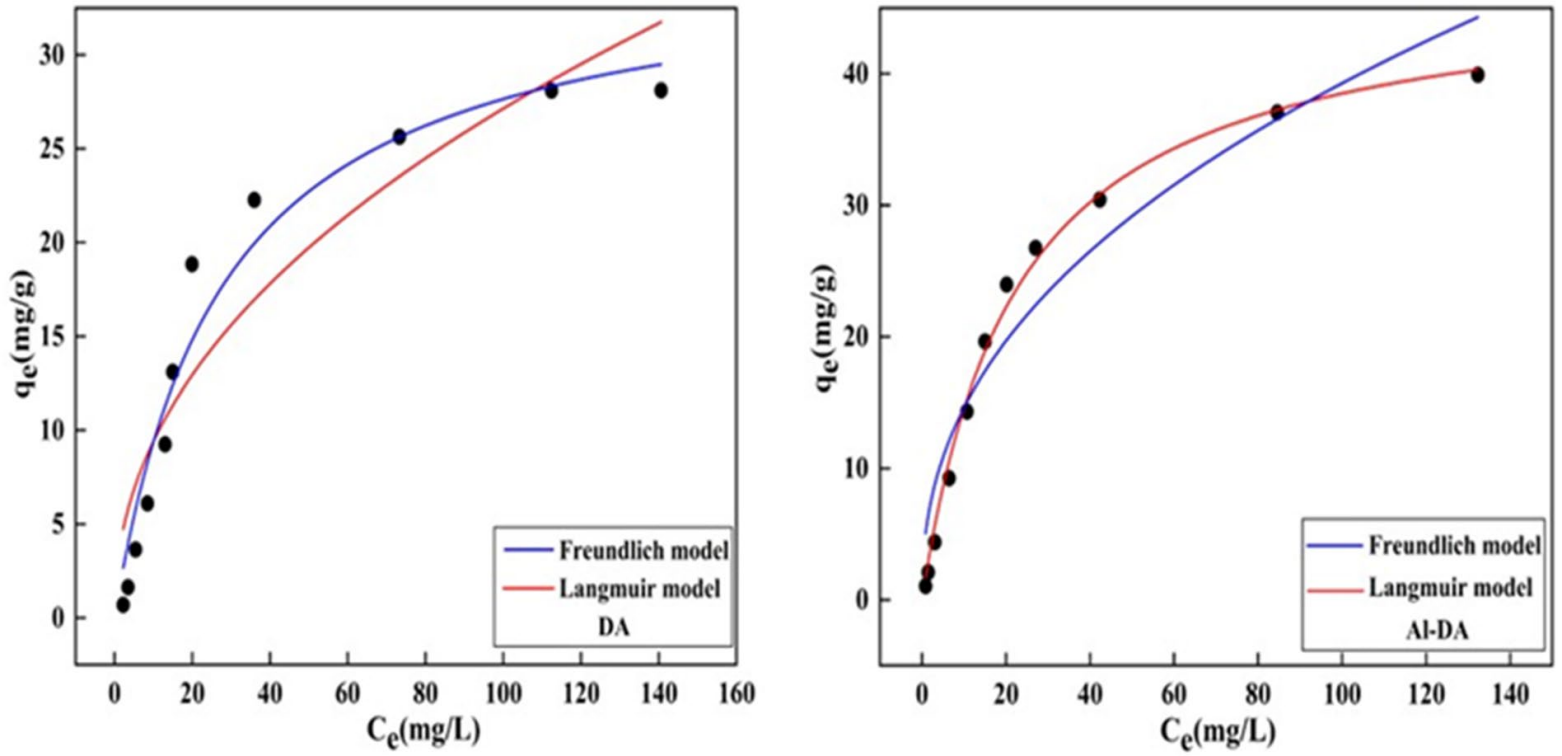
Adsorption isotherms of DA and Al-DA (pH 6, contact time 2 h, Sorbent dose 4 g/L).

The parameters of the isotherm model were obtained from Fig. 6 and are listed in Table 1. For the DA data, the Freundlich correlation coefficient (R^2^ = 0.9591) was larger than the Langmuir correlation coefficient (R^2^ = 0.8792), and the fitted curves yielded n values greater than 1, implying a strong affinity between the adsorbent and F^−^^40^. Thus, the adsorption process is not simple physisorption but stable chemisorption involving the formation of chemical bonds. The fits to the data for the adsorption of F^−^ by Al-DA show that both models effectively reflect the adsorption process. However, visual inspection of the fitted correlations shows that the Langmuir isotherm model can simulate the adsorption process of F^−^ on Al-DA more effectively than the Freundlich model. The adsorption mechanism is unimolecular layer adsorption that occurs mainly via ion-exchange interactions and is dominated by chemisorption. The maximum monolayer saturation adsorption capacity Q_m_ is 45.8050 mg/g. Similar results were reported in a previous study^6^. In addition, a large K_F_ reflects a strong binding ability^41^: K_F_ for Al-DA is larger than that of DA, indicating that Al-DA has a higher binding ability for F^−^. A comparison of the n and K_F_ values for DA and Al-DA indicates that Al-DA has a higher adsorption capacity for F^−^ than DA.

**Table 1:** Parameters of the isotherm models for F^−^ uptake by DA and Al-DA.

Adsorbent	Langmuir			Freundlich			RMSE
	Q_m_	K_L_	R^2^	K_F_	n	R^2^	
DA	32.5645	0.02851	0.8792	3.2575	1.8768	0.9591	0.1376
Al-DA	45.8050	0.04470	0.9949	6.5558	2.7038	0.9300	0.1256

### Adsorption kinetics

To further elucidate the adsorption mechanism, including the rate determination step, three kinetic models, the quasi-first-order kinetic, quasi-second-order kinetic, and intraparticle diffusion models, were used to investigate the adsorption of F^−^ by the adsorbents and the formula is as follows:

5 $${\text{ln}}\left( {{\text{q}}_{{\text{e}}} - {\text{q}}_{{\text{t}}} } \right){\text{ = lnq}}_{{\text{e}}} - {\text{K}}_{{\text{1}}} {\text{t}}$$

6 $$\frac{{\text{t}}}{{{\text{q}}_{{\text{t}}} }}{\text{ = }}\frac{{\text{1}}}{{{\text{K}}_{{\text{2}}} {\text{q}}_{{\text{e}}}^{{\text{2}}} }}{+}\frac{{\text{t}}}{{{\text{q}}_{{\text{e}}} }}$$

7 $${\text{q}}_{{\text{t}}} \;{ =}\;{\text{K}}_{{{\text{pi}}}} {\text{t}}^{{{{0.5}}}} {+ \text{C}}$$

where q_e_ is the adsorbed quantity at equilibrium (mg/g), q_t_ is the adsorbed quantity (mg/g) at the adsorption time t (min), and K_1_ is a quasi-first-order kinetic removal efficiency constant (min^−1^), K_2_ is a quasi-second-order kinetic removal efficiency constant (min^−1^), K_pi_ is an internal diffusivity constant, and C is a constant related to diffusion.

**Figure 7:**
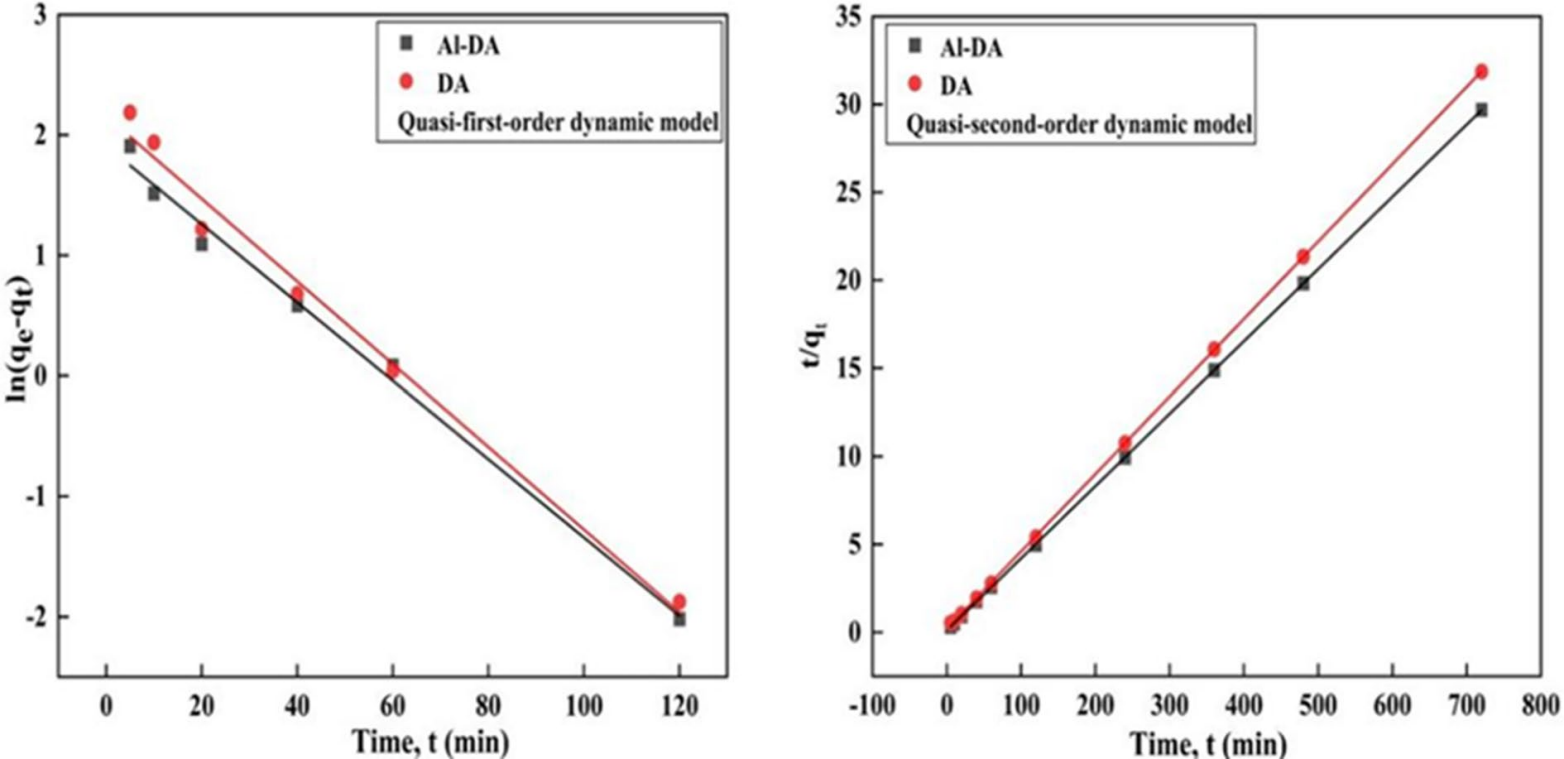
Fitting the experimental data into quasi-first-order and quasi-second-order kinetic equations (fluoride initial concentration is 100 mg/L, Sorbent dose 4 g/L, pH 6, contact time is 2 h).

The fitting results presented in Fig. 7 and Table 2 show that the quasi-secondary kinetic model effectively fits the data with quite high R^2^ values of 0.9998 and 0.9999. In addition, the theoretical adsorbed quantities obtained using the fitted quasi-second-order kinetic equations for DA and Al-DA data were closer to the experimental values than those obtained using the other models, indicating that the adsorption of F^−^ by DA and Al-DA is well described by the quasi-second-order kinetic model. This result suggests that chemical interactions between the absorbents and F^−^ control the adsorption process, chemisorption is the underlying mechanism. This type of chemical interaction usually corresponds to strong bonding between the surface functional groups of the adsorbent and the adsorbed species^37^. Therefore, F^−^ may form chemical bonds in the monolayer on the DA and Al-DA surfaces^42^. Xu^29^ also confirmed that the adsorption of fluorine by diatomite is effectively modeled by quasi-second-order kinetics.

**Table 2:** Quasi-first-order and quasi-second-order kinetic fitting results.

Adsorbent	Quasi-first-order kinetic model			Quasi-second-order kinetic model			RMSE
	q_e_	K_1_	R^2^	q_e_	K_2_	R^2^	
DA	3.6000	0.0182	0.9880	22.3221	0.0199	0.9998	0.05045
Al-DA	6.7500	0.0325	0.9924	24.3427	0.0168	0.9999	0.08757

**Figure 8:**
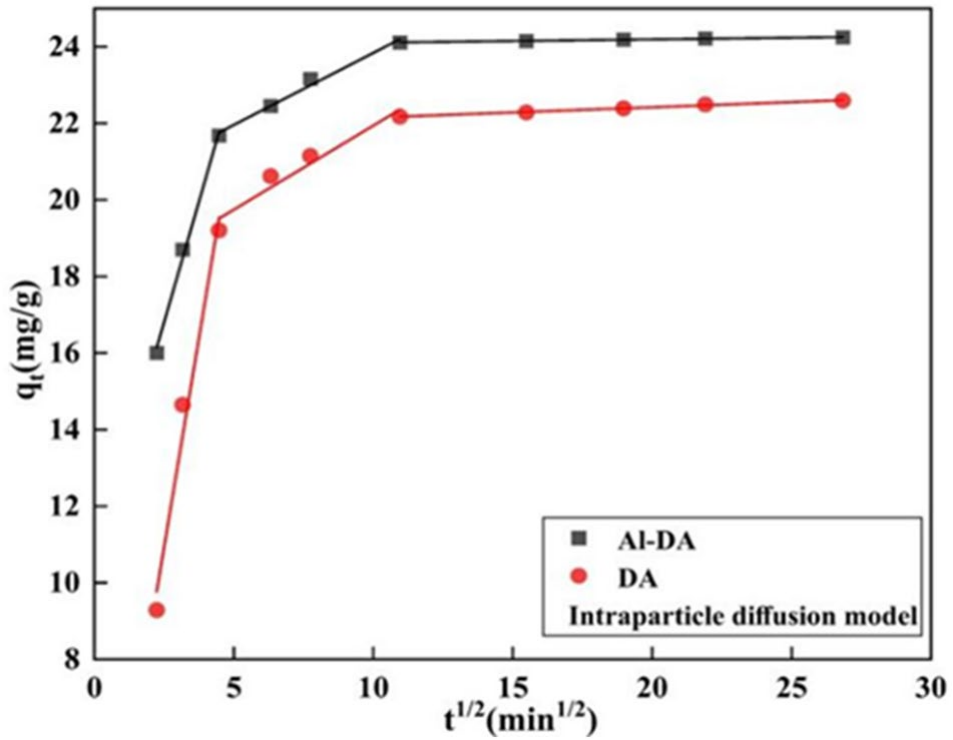
Fit the experimental data using the internal diffusion equation (fluoride initial concentration is 100 mg/L, Sorbent dose 4 g/L, pH 6, contact time is 2 h).

The adsorption of solid adsorbents can proceed by two types of adsorption, as well external and internal diffusion. As the type of diffusion that controls the removal efficiency differs during different phases of adsorption, the intraparticle diffusion model describes the transport of the target analyte from the aqueous phase to the surface of the adsorbent, followed by diffusion to the interior of the porous particles^43^. Therefore, the intraparticle diffusion model was used to study the characteristics of F^−^ adsorption, and the fitted results are shown in Fig. 8 and Table 3. The linear fit to the adsorption of F^−^ by DA and Al-DA consists of three segments with high individual R^2^ values, where K_p1_ > K_p2_ > K_p3_: the larger K_p_ is, the higher the diffusion rate is, indicating a two-step process for the adsorption of F^−^ by DA and Al-DA that conforms to the internal diffusion model. External diffusion occurs at the surface of the adsorbent, and the diffusion rate gradually decreases during the internal diffusion phase while ions diffuse into the interior of the adsorbent^44^. The data can be specifically interpreted as follows: the initial sharp region corresponds to external mass transport across a boundary layer; the second segment corresponds to gradual adsorption, where intraparticle diffusion is the rate-limiting step; and the third segment corresponds to equilibrium, during which intraparticle diffusion slows down^45,46^. Intraparticle diffusion is the rate-limiting step if the gradual adsorption segment passes through the origin. However, the fit to the data from this study does not pass through the origin, indicating that F^−^ adsorption on DA and Al-DA is controlled by both intraparticle diffusion and surface adsorption^46^.

**Table 3:** Internal diffusion model fitting results.

Adsorbent	K_p1_	R_1_^2^	K_p2_	R_2_^2^	K_p3_	R_3_^2^
DA	4.3724	0.9790	0.4390	0.9447	0.02710	0.9926
Al-DA	2.5206	0.9952	0.3760	0.9871	0.00863	0.9925

### Effect of different influencing factors on the adsorption of F^−^

#### Effect of the solution pH on the adsorption

**Figure 9:**
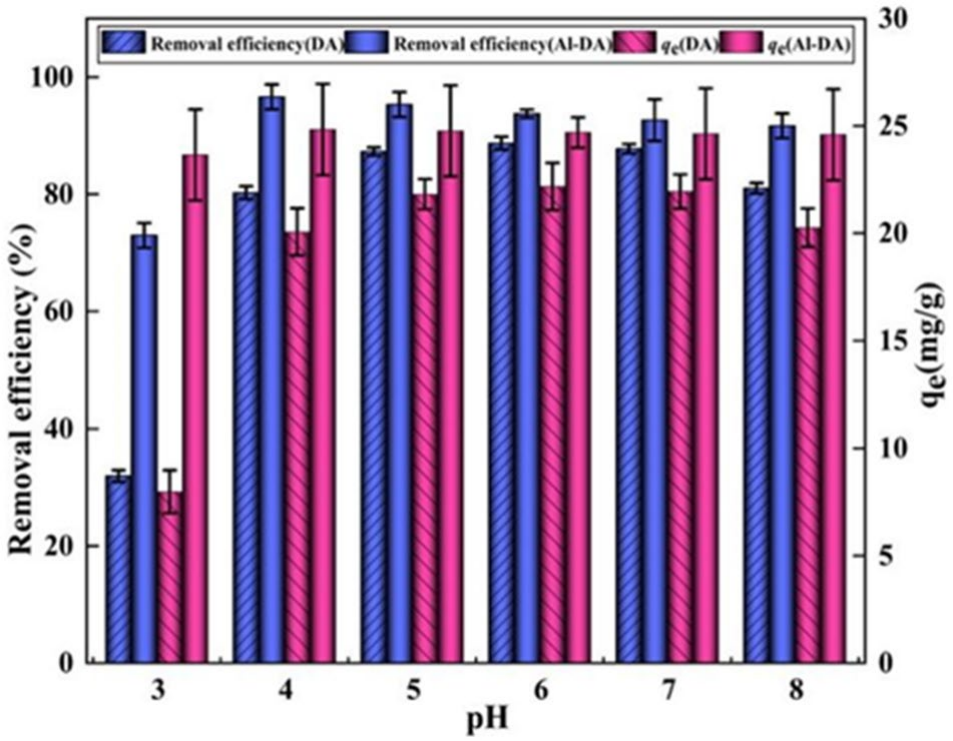
Effect of the pH on the adsorption of F^−^ by DA and Al-DA (fluoride initial concentration is 100 mg/L, Sorbent dose 4 g/L, contact time is 2 h).

The pH of the initial solution affects the solid–liquid interface of the adsorbent and the speciation of metal ions in the solution^47^. Therefore, it is crucial to investigate the effect of the initial pH on fluoride adsorption on DA and Al-DA. Therefore, adsorption experiments were carried out at different initial pH (4, 5, 6, and 7), and the results are shown in Fig. 9. The removal efficiency and adsorption capacity of F^−^ by both materials first increased with the pH and then decreased beyond a well-defined pH. For DA, the removal efficiency was 88.7% and the adsorption capacity was 22.2 mg/g at pH 6. For Al-DA, the removal efficiency was 97.0% and the adsorption capacity was 24.8 mg/g at pH 4. Subsequently, with the increase in pH, the removal efficiency and adsorption capacity of DA and Al-DA on F^−^removal efficiency and adsorption capacity gradually decreased with the increase in pH. Acidic conditions result in a high number of H^+^ on the adsorbent surface, resulting in electrostatic attraction between the positively charged adsorbent surface and F^−^. It has also been found that the removal efficiency and adsorbed quantity of F^−^ can be significantly increased by the formation of Al-F complexes^6,27^. With increasing pH, F^−^ and OH^−^ compete for adsorption sites, where the higher the OH^−^ concentration is, the more significant the competitive adsorption effect is, leading to a decrease in the removal efficiency and adsorption capacity of F^−^^41^. The investigated materials have good fluoride removal performance over a wide pH range, but as a higher fluoride removal capacity can be obtained in acidic media than in alkaline media, the optimal pH for adsorption of F^−^ by DA and Al-DA are 6 and 4, respectively. Because Al-DA is modified with aluminum salts and some cations are introduced, resulting in an increased positive charge, the optimal pH for the adsorption of fluoride ions will be different from that of DA, and Al-DA has to show a better adsorption effect.

### Effect of the dosing quantity of the adsorbent on the removal efficiency and capacity

**Figure 10:**
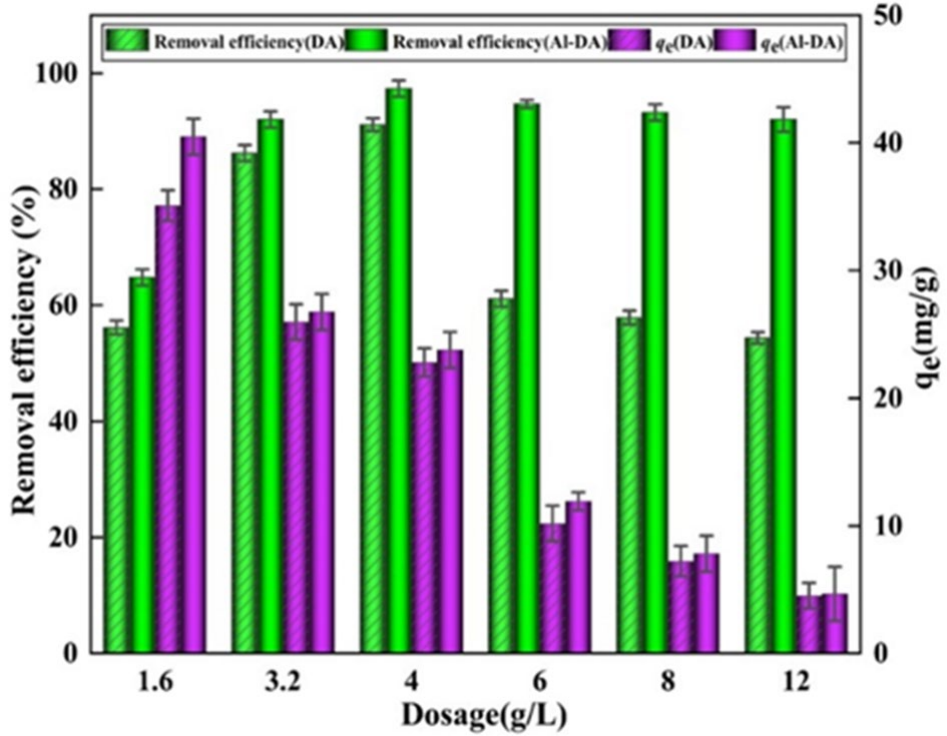
Effect of the dosing quantity of DA and Al-DA on the adsorption of F^−^ (fluoride initial concentration is 100 mg/L, pH 6, contact time is 2 h).

Figure 10 shows the effect of using masses of the two adsorbents ranging from 1.6 to 12 g/L on F^−^ adsorption. The removal efficiency of F^−^ on DA and Al-DA increased with the dosage to maxima of 91.1% and 97.3% at a dosage of 4 g/L, respectively, and then started to decrease. Increasing the adsorbent dose corresponds to increasing the surface area and the number of adsorption sites available for the same quantity of fluoride ions. Therefore, the removal efficiency of F^−^ increased considerably with the dose during the initial stage^6,48^. This finding is consistent with reports of the effect of the adsorbent dosage on fluoride removal^49,50^. The subsequent decrease in the adsorption capacity resulted from an increase in the fixed initial fluorine concentration and the solid dose of the fixed solute load, which led to a decrease in the availability of fluoride ions per unit mass of adsorbent^36^. Therefore, the analysis shows an optimal dose of 4 g/L for both DA and Al-DA, and higher adsorption of F^−^ by Al-DA than by DA.

### Effect of coexisting anions on the adsorption of F^−^

**Figure 11:**
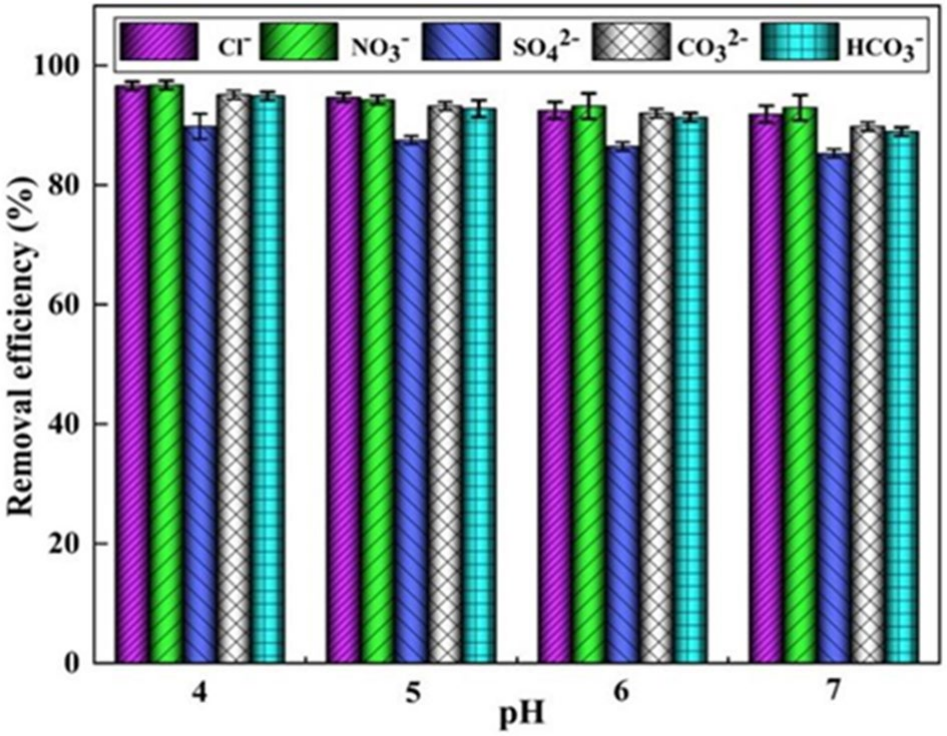
Effect of anions on F^−^ adsorption on Al-DA as a function of the pH (ion concentration is 100 mg/L, Sorbent dose 4 g/L, contact time is 2 h).

Fluorinated natural water and wastewater typically contain a variety of anions, such as Cl^−^, NO_3_^−^, SO_4_^2−^, CO_3_^2−^, and HCO_3_^−^. During adsorption, these coexisting anions may compete with F^−^ for adsorption sites, thus reducing the removal efficiency of the adsorbent. The competitiveness for the adsorption of a coexisting anion depends on the relative concentration of the ion and its affinity for the adsorbent, which is intrinsically related to the ionic radius and charge^41^. Therefore, it is crucial to assess the effect of coexisting ions on the uptake of F^−^ for practical applications. Figure 11 shows the effect of the presence of Cl^−^, NO_3_^−^, SO_4_^2−^, CO_3_^2−^, and HCO_3_^−^ on F^−^ adsorption at different pHs. The highest removal efficiency was observed at pH = 4, and the interference capacity de-creased in the order SO_4_^2−^  > HCO_3_^−^  > CO_3_^2−^  > Cl^−^  > NO_3_^−^, which is consistent with the results of the previous studies^6,29^. However, all the anions have a limited ability to inhibit the adsorption of F^−^. For example, at pH = 4, the adsorption of F^−^ reaches 89.8% even under the interference of SO_4_^2−^. Therefore, it can be speculated that Al-DA has a high affinity for F^−^.

### Fluoride removal from a real-life water sample

To examine the efficiency of the prepared adsorbent for the removal of fluoride from the real field sample, we collected water samples from a sewage treatment station of an enterprise in the fluorine chemical industrial park. The dosage was 4 g/L, the thermostatic adsorption experiment was carried out, and the effect of removing fluoride ions from actual wastewater was compared with that reported in other literature. The results are shown in Table 4. After the adsorption study, the fluoride concentrations were well below the specified limit according to WHO (< 1.5 mg/L). Compared with other adsorbents, Al-DA can reduce the concentration of fluoride ions to a relatively low safety value. Al-DA shows good advantages. Therefore, the Al-DA adsorbent developed in this study can be used to remove real fluoride-polluted water.

**Table 4:** Fluoride removal study from the real-life water sample.

Adsorbent	F^−^ concentration before adsorption (mg/L)	F^−^ concentration after adsorption (mg/L)	Removal efficiency (%)	References
Fe-Al nanocomposite	4.2	0.75	82.14	^16^
CeBC-A@CS	2.08	0.63	69.71	^51^
Zn-Fe-Ch	14	1.38	90.14	^52^
HNAA	17.5	0.84	95.20	^39^
Al-DA	5.64	0.72	87.23	Present work

## Conclusions

Given the affinity between aluminium, a Lewis acid electron acceptor, and F^−^, a Lewis base electron donor, the potential of using DA as a raw material and modifying DA with aluminium hydroxide as an adsorbent for fluoride removal from the water was investigated in this study. The results showed that aluminium hydroxide was successfully encapsulated by DA. The adsorption mechanism of F^−^ on DA and Al-DA mainly involves electrostatic attraction, complexation, and ion-exchange processes, as illustrated by changes in the SEM, EDS, XRD, and FTIR results for the adsorbents before and after adsorption, as well as by zeta potential analysis, isothermal fitting, and kinetic fitting analysis. Adsorption experiments showed an increase in the adsorption efficiency of F^−^ from 91.1 to 97.3% by modifying DA with aluminium hydroxide. The optimum dosage of both DA and Al-DA was 4 g/L, and the optimum pH for F^−^ adsorption by DA and Al-DA were 6 and 4, respectively. The adsorption of F^−^ by Al-DA was selective even in the presence of competing ions. The results of this study demonstrate the potential of alumina-hydroxide-modified DA as an effective adsorbent for the defluoridation of wastewater.

## Data Availability

All data generated or analyzed during this study are included in this published article.
